# Proceedings of the Academy of Medicine and Surgery Richmond, Va.

**Published:** 1891-03

**Authors:** James N. Ellis

**Affiliations:** Reporter; Richmond, Va.


### PROCEEDINGS OF THE ACADEMY OF MEDICINE AND SURGERY.

Richmond, Va., Jan. 13th, 1891.

Dr. Chas. M. Shields, President, in the chair.

Following the reading of Dr. Tulner's paper on "Hour-glass Contraction with Retained Placenta," Dr. W. W. Parker referred to some experiences with retained placenta, and advised a speedy examination and immediate removal of the after-birth, upon the completion of the second stage of labor. He considers such minute and careful attention to the cleansing and disinfection of the hands as unnecessary and superfluous.

Dr. Edward McCarthy asked if it was the opinion of the members of the Academy that the use of chloroform during labor subjected the patient to greater risk of hemorrhage. In seeming opposition to this view he cited a case, reported by Dr. W. T. Oppenheimer at a previous meeting, in which haemoptysis was promptly arrested by the inhalation of chloroform. He had himself observed its haemostatic effect when directly applied to a bleeding surface.

Dr. Jno. N. Upshur could recall but two cases of hour-glass contraction in his experience. The first one occurred in the first year of his professional life, and the readiness with which it was relieved leads him to doubt the correctness of his diagnosis. The second case occurred in a negro woman, and was attributed to the unadvised administration of a full dose of ergot by a midwife. The band of constricting muscular fibers, in a state of clonic spasm, was midway between the fundus and ostium internum, and from one to two inches broad. Inserting two fingers within this opening, through which the blood was escaping in a sluice, he succeeded in dilating the constriction sufficiently to admit of the removal of the placenta. The uterus promptly contracted, and the hemorrhage ceased, but she died from exhaustion in a few hours. He thinks we should wait a reasonable length of time—sufficient to tie the cord, care for the child, &c.—before attempting to deliver the placenta, combining Crede's method with gentle traction, and using the finger to assist in securing complete detachment of the placenta. It is his habit to give a dose of ergot just as the head passes the ostium vaginae, especially if chloroform has been administered, as its use undoubtedly predisposes to hemorrhage. He once used chloroform continuously for several hours in a case of protracted labor, consequent upon premature ossification of the cranial sutures. Ergot was given, the labor finally completed, and when the doctor left at 12 o'clock the womb was well contracted. She lost some blood during the night, but firm contraction was again obtained before leaving her in the morning. While sitting by her bed that night, (20 hours after delivery) there was a sudden and alarming gush of blood. Four ounces of the per-sulphate of iron dissolved in one quart of hot water was applied, by means of a fountain syringe, to the uterine cavity; the womb contracted, the hemorrhage ceased, and there was no further trouble. The doctor attributes the bleeding to the long continued use of chloroform, and thinks this tendency of the drug (to predispose to hemorrhage) the most effectual barrier to its habitual employment in obstetrical work. He has also known it to arrest uterine contractions to such an extent that the labor did not proceed until the patient was allowed to come from under its influence.

The doctor advocates a medium course in the matter of antisepsis. He does not think it necessary to subject the patient to the discomfort of antiseptic injections several times daily after a clean labor—but has the vulva sponged with hot water two or three times a day, using a soft napkin and a little vaseline, but avoiding grease, the heat of the body causing such substances as lard to become rancid, irritant and septic. If there is much soreness a douche of hot water containing borax will prove of benefit, but all vaginal douches are attended with danger, and the speaker has met with cases of uterine colic and fatal peritonitis resulting from their use.

Dr. Landon B. Edwards said that his worst case of post-partem hemorrhage occurred in a patient who was delivered without chloroform, and he thinks it is not always responsible for the hemorrhages following its use. He used it with benefit in the only important case of hour-glass contraction he has ever encountered. Other means referred to by the doctor as available in the treatment of hour-glass contraction were the use of the calpeurynter, belladonna, and hot water injections. Vary the treatment according to the indications of the particular case. Time is an element of great importance. *Wait.* There is no special danger of hemorrhage in cases of fundal implantation, if the uterus is contracted, as the retained placenta acts as a tampon above the constricting band.

Dr. Upshur suggested that the uterus might be released *above* the contraction ring, and hemorrhage occur as in the case above cited by him.

Dr. J. S. Wellford thinks that the great majority of cases diagnosed hour-glass contraction are simply cases of retained placenta, such as where the upper part of the fundus is contracted, and firmly clasping the placenta. He would here give chloroform, and introducing the hand, bring down the afterbirth. Ordinarily he succeeds in delivering the placenta without introducing the hand, by compressing the fundus through the abdominal walls and making gentle traction upon the cord. After the child is born the womb, completely exhausted, has a period of physiological rest preparatory to the completion of labor by the expulsion of the placenta; and it is well to permit this period to elapse before attempting its removal. Time the administration of ergot, so that its maximum effect may be experienced at the moment the head passes the labia.

Dr. Upshur is satisfied of the utility of chloroform in the treatment of hour-glass contraction, but thinks it inferior to nitrite amye or nitro-glycerine. The effects of the latter, when administered by the mouth, in doses of 1-50th of a grain, are experienced in six minutes, effectually relieving uterine spasm.

Dr. W. W. Parker has seen four or five cases of undoubted hour-glass contraction, in which there was no possibility of making a mistake as to diagnosis. He always uses chloroform in his labor cases and has never had a case of fatal post-partum hemorrhage. He personally experienced entire relief from its use, in an incipient attack of nephritic colic; and has seen it cut short an incipient fever when given by the mouth.

Dr. M. D. Hoge, Jr., has seen it given by the mouth, in half teaspoonful doses, repeated until general anaesthesia sufficient for surgical operations was induced.

Dr. J. S. Wellford has found it an excellent remedy, given in teaspoonful doses, with milk as a vehicle, in attacks of colic. For internal administration he uses spiritus chloroform, in which form it is miscible with water.

Dr. Geo. Ben Johnston reported the following case:

Twenty months ago he saw, in consultation with Dr. James B. McCaw, a woman who had been suffering for some time with some pelvic trouble. She was 30 years old, the mother of two children, in good health previous to her marriage. Since that time, however, she had been subject, at irregular periods, of copious pulmonary hemorrhage, excited by the most ordinary kinds of exercise, and with no evidences of pulmonary lesions discoverable by auscultation or percussion. This finally subsided, but was followed by intermittent pains in the pelvic regions, which were increased by exercise. There was some swelling in the ovarian region, but the menses were regular, and her general health good. There were occasional discharges from the vagina of from 1-2 to 2 teacupsful, of an ascitic like fluid, accompanied with, or followed by a complete but temporary cessation of pain. A cyst of the ovary communicating with a fallopian tube, through some obstruction of which the accumulating fluid occasionally forced a passage, was diagnosed. The pains finally became so intolerable that morphia had to be used freely, and it being apparent that she would soon become a confirmed opium eater, it was decided to operate. This was accordingly done in July, 1889. The left ovary was found to contain the remains of a collapsed cyst; the right ovary a beginning cyst, and both tubes were hydropsed. The ovaries and tubes were removed, and the patient made an uneventful and good recovery. But presently she was seized by an epiliptiform attack, falling, foaming at the mouth, &c. She was treated for this, but after eight or nine days they returned and progressively increased in frequency until they were of daily occurrence. Under the skillful treatment of Dr. McCaw, these attacks finally ceased, but were followed by pains which she described as being located between the entrails and back, and so severe that she again resorted to opium, which was gradually increased until she was taking five grains of the sulphate of morphia hypodermically each day. Appetite poor and capricious. During the last six months of her sickness there was a recurrence of those peculiar vaginal discharges, but for the three months immediately preceding she has been free from them. It was now determined to make an exploratory operation; and this was done a week ago today, the incision being made through the cicatrix resulting from the former operation. The abdomen being opened, an exploration revealed nothing to account for the pain and fluid. The stumps of the tubes had healed in a satisfactory manner, the uterus shrunken to 1-4 its former size, the shrinkage not being uniform, but presenting somewhat of an hour-glass shape. Failing to find sufficient cause here for her troubles, a further investigation of the contents of the abdominal cavity was instituted, which discovered the appendix virmiformis distended by a number of firm bodies, the size of an ordinary black pea. Lifting the appendix out of the wound, the neighboring portions of the gut were seen to be somewhat injected, but not inflamed. The appendix was then amputated, and after the toilet of the peritonium was completed, a rubber drainage tube, dipping down to the colon, was inserted, and the abdominal wound closed. Reaction was prompt, temperature is normal, and her recovery seems now assured. The drainage tube was removed the second day. The wound has apparently united by first intention, and the normal temperature and good character of the pulse, are considered as sufficient to justify him in permitting the dressing to remain undisturbed until to-morrow.

The doctor does not report this case as a cure. Time alone will determine that. But the cessation of pain following the removal of the appendix leads him to hope so; and he will report the result at some subsequent meeting. He then submitted the amputated appendix to the inspection of the members. It was found to contain some seven or eight little bodies of a semi-solid consistency, emitting a faeculent odor. These will subsequently be subjected to a chemical and microscopical examination.

JAMES N. ELLIS, M. D., Reporter.

